# Lipoprotein(a) and calcific aortic stenosis: from inherited risk marker to therapeutic target

**DOI:** 10.3389/fmed.2026.1908850

**Published:** 2026-08-05

**Authors:** Francisco Epelde

**Affiliations:** 1 Internal Medicine Department, Parc Taulí Hospital Universitari, Institut d'Investigació i Innovació Parc Taulí (I3PT-CERCA), Universitat Autònoma de Barcelona, Sabadell, Spain; 2 Internal Medicine Department, APTIMA Centre Clinic, Mútua de Terrassa, Terrassa, Spain

**Keywords:** aortic stenosis, aortic valve replacement, apolipoprotein(a), autotaxin, calcific aortic valve disease, lipoprotein(a), LPA, olpasiran

## Abstract

**Background:**

Calcific aortic valve stenosis (CAVS) is the most common valvular heart disease in older adults and remains a condition for which valve replacement is the only established disease-modifying treatment. Converging genetic, epidemiological, imaging and mechanistic evidence supports an active disease process involving lipid deposition, inflammation, extracellular matrix remodeling, osteogenic differentiation and progressive mineralization. Lipoprotein(a) [Lp(a)] has emerged as an inherited risk factor of particular interest because it links apolipoprotein(a), apolipoprotein B, oxidized phospholipids (OxPL), autotaxin (ATX)-lysophosphatidic acid signaling and valvular calcification.

**Objective:**

To review the evidence connecting Lp(a) with the initiation and progression of CAVS, distinguish levels of evidence and plausible mediators, and discuss the current and future clinical implications of Lp(a) measurement and Lp(a)-lowering therapy in aortic valve disease.

**Methods:**

A narrative review was performed using PubMed/MEDLINE, major cardiovascular guidelines, consensus statements and reference lists of key articles. Searches were updated to June 2026 and prioritized genetic studies, Mendelian randomization analyses, prospective cohorts, imaging studies, tissue and mechanistic studies, systematic reviews, randomized trials and early-phase pharmacological studies relevant to Lp(a), oxidized phospholipids and CAVS.

**Results:**

Genetic and Mendelian randomization studies support a likely causal contribution of the LPA locus and lifelong exposure to apoB-containing lipoproteins to aortic valve calcification and incident CAVS. Clinical and imaging studies associate higher Lp(a) concentrations with prevalent valve calcification, incident aortic stenosis and, in selected cohorts, faster haemodynamic progression. Mechanistically, Lp(a)-associated particles and lipid mediators have been detected in valve tissue, and OxPL, autotaxin-derived lysophosphatidic acid and related inflammatory pathways can promote valve interstitial cell osteogenic transformation. However, evidence for progression is heterogeneous, and established calcific disease is also shaped by baseline calcium burden, valve anatomy, renal-mineral pathways, inflammation, fibrosis and mechanical stress. Existing statin-based and mineral-targeted therapies have not slowed established CAVS, while potent Lp(a)-lowering agents can markedly reduce Lp(a) but still lack definitive valvular outcome data.

**Conclusions:**

Lp(a) should be considered a cardiovascular risk enhancer and a plausible causal contributor to calcific aortic valve disease, especially during earlier lipid-inflammatory and microcalcific phases. Measuring Lp(a) at least once in adulthood is supported by contemporary lipid guidance and may be particularly informative in premature CAVS, family clustering, coexisting premature atherosclerotic cardiovascular disease or unexplained rapid progression. Whether pharmacological Lp(a) lowering can prevent CAVS or slow progression remains an important but unproven translational question.

## 1 Introduction

Calcific aortic valve stenosis (CAVS) is a major cause of morbidity, mortality and health-care utilization in aging societies. Its clinical trajectory is familiar: a long silent phase of aortic sclerosis and valve calcification is followed by progressive obstruction, left ventricular pressure overload, symptoms and eventual need for surgical or transcatheter valve replacement. The biological interpretation of this process has changed substantially. CAVS is no longer regarded as simple mechanical wear or passive calcium deposition. It is increasingly understood as an active disease continuum involving lipid infiltration, endothelial dysfunction, inflammation, extracellular matrix remodeling, osteogenic signaling and progressive mineralization (1–6).

Despite this biological progress, CAVS remains unusual among common cardiovascular diseases because no drug has convincingly been shown to halt or reverse its progression once clinically significant stenosis is established. Aortic valve replacement, either surgical or transcatheter, remains the definitive therapy for severe symptomatic disease or for severe disease with adverse ventricular or haemodynamic features according to professional guidelines (7, 8). This therapeutic gap has encouraged renewed attention to upstream mechanisms and to the possibility that earlier intervention, before dense calcification becomes self-propagating, may be required.

Among lipid-related factors, lipoprotein(a) [Lp(a)] has emerged as a particularly attractive candidate. Lp(a) is an LDL-like particle composed of apolipoprotein B-100 covalently bound to apolipoprotein(a), a highly polymorphic glycoprotein encoded by the LPA gene. Circulating concentrations are largely genetically determined, remain relatively stable throughout life and are only modestly influenced by lifestyle or most conventional lipid-lowering therapies (9–13). Lp(a) may promote risk through overlapping properties: it is an apoB-containing lipoprotein capable of tissue lipid deposition; it is enriched in oxidized phospholipids (OxPL), which trigger inflammatory and osteogenic pathways; and its apo(a) component is structurally homologous to plasminogen, potentially influencing fibrinolysis and thrombosis (14, 15).

The relevance of Lp(a) to CAVS was reinforced by genetic studies linking variants at the LPA locus with aortic valve calcification and incident aortic stenosis (16–19). Subsequent observational, imaging and tissue studies have shown associations among Lp(a), OxPL, active valve calcification and haemodynamic progression (20–31). The field has now entered a translational phase: potent Lp(a)-lowering therapies are in clinical development, including antisense oligonucleotides, small interfering RNA agents and oral inhibitors of Lp(a) assembly (32–42). These therapies were initially developed to reduce residual atherosclerotic cardiovascular risk, but their implications for valvular disease may be equally important.

This review summarizes the relationship between Lp(a) and CAVS, focusing on biological plausibility, levels of evidence, imaging correlates, clinical implementation, therapeutic implications and unresolved questions. The central argument is that Lp(a)-associated CAVS is a plausible model for precision prevention in valvular heart disease, while acknowledging that valve-specific outcome data for Lp(a)-lowering therapy remain pending.

## 2 Methods: narrative-review strategy and evidence interpretation

This is a narrative review rather than a systematic review or meta-analysis. PubMed/MEDLINE was searched up to June 2026 using combinations of the following terms: “lipoprotein(a)”, “Lp(a)”, “LPA”, “calcific aortic valve disease”, “aortic stenosis”, “aortic valve calcification”, “oxidized phospholipids”, “OxPL”, “autotaxin (ATX)”, “lysophosphatidic acid”, “valve interstitial cell”, “Mendelian randomization”, “genome-wide association”, “aortic valve replacement”, “pelacarsen”, “olpasiran”, “zerlasiran”, “lepodisiran” and “muvalaplin”. Major cardiovascular guidelines, lipid-society consensus statements and reference lists of key articles were also reviewed.

Eligible evidence included guidelines, consensus documents, genetic association studies, Mendelian randomization analyses, prospective and retrospective cohorts, imaging studies, tissue and *in vitro* mechanistic studies, systematic reviews, randomized trials of lipid or mineral pathways in CAVS, and early-phase pharmacological studies of Lp(a)-lowering therapies. Priority was given to peer-reviewed publications, prospective designs, larger cohorts, clinically relevant endpoints and studies directly addressing Lp(a), OxPL, autotaxin or valve calcification. Seminal older studies were retained when necessary to explain mechanistic plausibility or prior therapeutic failures.

Because the evidence base includes heterogeneous study designs, the revised manuscript distinguishes levels of evidence throughout. Genetic and Mendelian randomization studies provide the strongest support for causal inference, but they model lifelong exposure and do not establish that short-term pharmacological lowering will modify established valve disease. Observational and imaging cohorts provide clinical and biological associations but remain susceptible to confounding, selection bias, endpoint heterogeneity and differences in assay methods. Tissue and *in vitro* studies establish plausibility and candidate mediators but require clinical validation. Early-phase drug trials demonstrate Lp(a)-lowering efficacy but do not yet provide definitive CAVS outcome evidence. Table 1 summarizes the principal evidence domains and their interpretation.

**Table 1:** Key evidence linking Lp(a) with calcific aortic valve disease.

Evidence domain	Representative evidence	Main finding	Interpretation
Genetics	Thanassoulis et al. (16), Kamstrup et al. (17), Arsenault et al. (18)	LPA variants and high Lp(a) are associated with aortic valve calcification and incident CAVS	Supports likely causal contribution and a lifelong-exposure model
Population cohorts	Copenhagen cohorts; MESA; multiethnic studies (17, 28–30)	Elevated Lp(a) is associated with prevalent valve calcification and clinical valve disease	Measurement may identify inherited valve risk, especially in premature disease
Valve tissue and mechanisms	O'Brien et al. (61), Torzewski et al. (62), Yu et al. (63), Zheng et al. (21)	ApoB/apo(a)-containing material, Lp(a)-associated molecules and OxPL-related pathways are present in diseased valves and promote VIC calcification	Provides biological plausibility beyond epidemiological association
Autotaxin and lipid mediators	Bouchareb et al. (64), Bourgeois et al. (65), Yoon et al. (66), Cayer et al. (67)	ATX-LPA signaling and oxylipin/prostaglandin pathways may modulate inflammation, fibrosis and mineralization	Lp(a)-related biology sits within a broader lipid-inflammatory network
Imaging	CT calcium scoring; 18F-NaF PET studies (23, 43, 54–56)	CT quantifies calcific burden; PET identifies active microcalcification	Imaging can enrich trials and provide intermediate endpoints
Longitudinal progression	Capoulade et al. (22), Zheng et al. (21), Kaiser et al. (26), Arsenault et al. (57), Girard et al. (20)	Progression associations are heterogeneous but higher Lp(a) is linked with faster haemodynamic progression in selected analyses	Supports trials while emphasizing stage and endpoint dependence
Therapeutic inference	Statin/mineral trials and emerging Lp(a)-lowering studies (32–46, 58, 69–72)	Late LDL/mineral strategies have not slowed CAVS; new agents markedly lower Lp(a)	Valve-specific outcome trials are required before therapeutic claims

## 3 Calcific aortic stenosis as an active lipid-inflammatory disease

CAVS develops across a continuum, beginning with aortic sclerosis and microscopic calcific deposits and progressing toward leaflet thickening, restricted cusp motion and haemodynamically significant obstruction. Histologically, early lesions resemble atherosclerosis in several respects: endothelial disruption, subendothelial lipid deposition, inflammatory cell recruitment, oxidative stress and extracellular matrix remodeling. As disease advances, valve interstitial cells (VICs) acquire osteoblast-like features and deposit hydroxyapatite. The valve becomes progressively stiffer, and the haemodynamic burden accelerates calcification through mechanical stress and maladaptive repair (2–5, 43).

Traditional risk factors for CAVS overlap with those for atherosclerosis, including age, male sex, hypertension, smoking, chronic kidney disease, diabetes and dyslipidaemia (1–5). However, CAVS is not simply coronary atherosclerosis of the valve. The aortic valve is a biomechanically specialized organ exposed to repetitive shear stress, bending, stretch and flow turbulence. Leaflets have distinct fibrosa, spongiosa and ventricularis layers, each with different cellular and extracellular matrix properties. These features influence where lipids enter, where inflammatory cells accumulate and where calcium nodules develop (2–5, 43). The local mechanical environment also helps explain why calcification may accelerate after early disease has been established.

This timing issue is central to the apparent paradox in the field: lipids are strongly implicated in CAVS biology, yet lipid-lowering trials with statins did not slow established stenosis (44–46). Rather than excluding lipid causality, these trials suggest that the therapeutic window may be earlier than the stage at which patients typically enter clinical trials. Once macroscopic calcium, leaflet rigidity and altered stress distribution are present, the disease may become less dependent on lipid entry and more dependent on osteogenic, fibrotic and mechanical feedback loops. Lp(a) is particularly relevant to this model because it may act upstream, promoting lipid retention, inflammatory activation and microcalcification before severe obstruction is clinically apparent.

The strongest lipid evidence comes from genetics. Variants associated with LDL-C, apoB and familial hypercholesterolaemia have been linked with aortic valve calcification and incident CAVS (47–49). More specifically, genetic variation in LPA has been associated with aortic valve calcification and stenosis, suggesting that Lp(a) is not merely a marker of risk but a likely causal participant in the disease process (16–19).

## 4 Biology of Lp(a): particle effects, OxPL cargo and the autotaxin axis

Lp(a) is structurally similar to LDL, but with the distinctive addition of apo(a), which is covalently attached to apoB-100. Apo(a) contains kringle domains homologous to those in plasminogen; the number of kringle IV type 2 repeats varies widely and is inversely related to plasma Lp(a) concentration. This genetic architecture explains why Lp(a) concentrations differ substantially among individuals and ancestries and why a single measurement generally captures lifelong exposure better than many other lipid biomarkers (9–13, 50, 51).

Several features make Lp(a) relevant to the aortic valve. First, tissue studies have demonstrated that apolipoproteins B and (a) accumulate in early and end-stage aortic valve lesions, supporting the presence of apoB/apo(a)-containing material within diseased valve tissue rather than only in plasma (61). Second, Lp(a)-associated molecules, including OxPL and lysophosphatidic acid, have been detected in plasma and valve leaflets from patients with CAVS (62). Third, Lp(a) is a major carrier of OxPL, which can stimulate endothelial activation, monocyte recruitment, cytokine release and VIC osteogenic differentiation (14, 21, 22, 62, 63). Fourth, the apo(a) component may contribute to antifibrinolytic or prothrombotic biology, although the clinical importance of this pathway in CAVS remains less certain (15, 24).

The autotaxin (ATX)-lysophosphatidic acid pathway provides an additional mechanistic bridge between Lp(a), OxPL and mineralization. ATX is a lysophospholipase D that converts lysophosphatidylcholine into lysophosphatidic acid, a bioactive lipid mediator with inflammatory, fibrotic and osteogenic effects. ATX can be transported by Lp(a), can be secreted by VICs, and has been implicated in inflammation and mineralization of the aortic valve (64, 65). Experimental work also suggests that ATX inhibition can attenuate fibro-calcific remodeling of VICs and aortic valve calcification in preclinical models (66). These data support the biological plausibility of ATX as a modulator of Lp(a)-related valve injury, while not yet establishing a clinical indication for ATX-targeted therapy.

A key conceptual point is whether Lp(a) itself is directly pathogenic or whether much of its biological activity is mediated by cargo such as OxPL and by downstream ATX-lysophosphatidic acid signaling. The most balanced interpretation is that these mechanisms are intertwined. The Lp(a) particle provides an apoB-containing structure capable of tissue entry and retention and an apo(a) scaffold that influences particle metabolism, inflammatory cargo and possibly fibrinolysis. OxPL carried on Lp(a) appear to be major effectors of inflammatory and osteogenic signaling, and *in vitro* work suggests that inactivating OxPL can attenuate Lp(a)-induced VIC calcification (21, 63). ATX further converts lipid substrates into lysophosphatidic acid, amplifying inflammation, fibrosis and mineralization (64–66).

**Figure 1:**
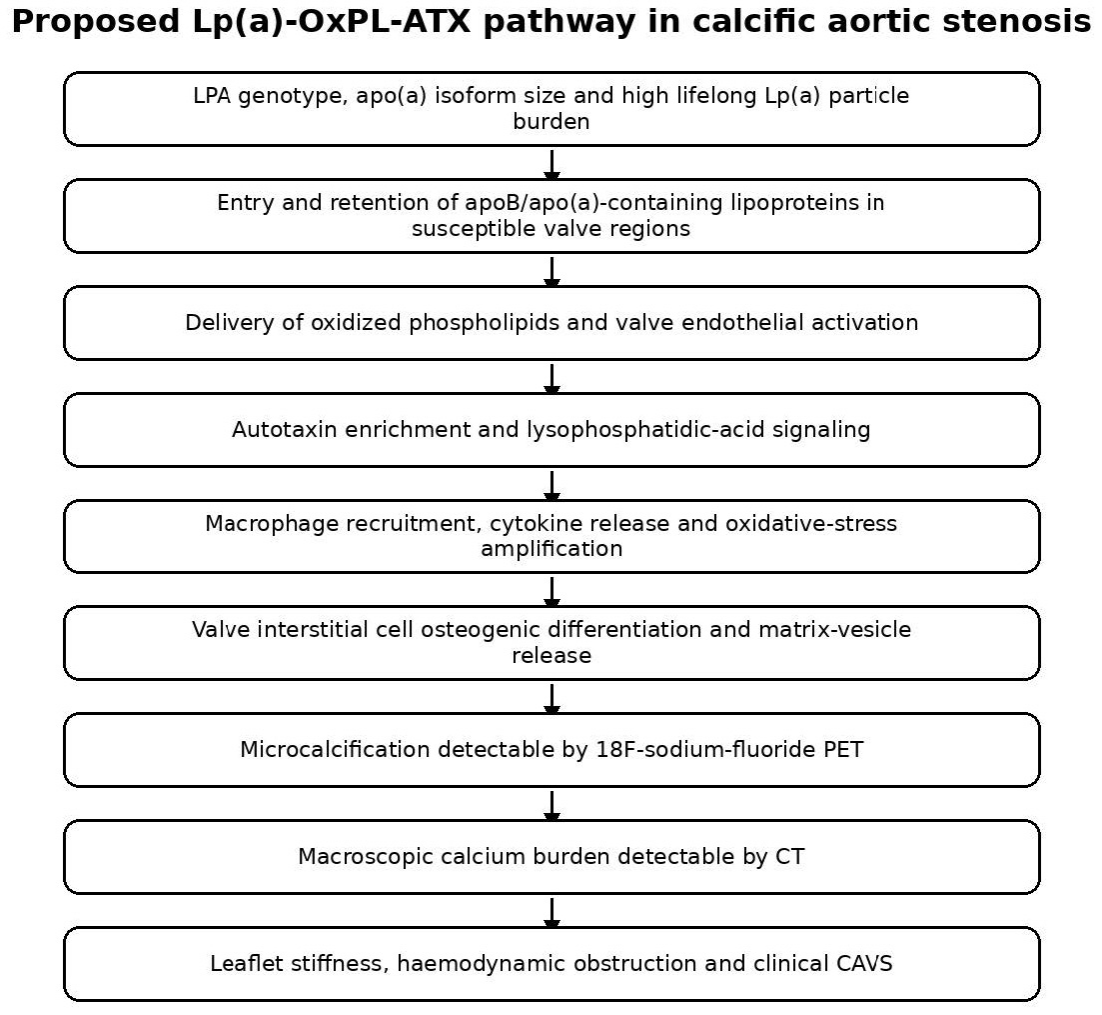
Proposed Lp(a)-OxPL-ATX pathway in calcific aortic stenosis. The pathway is most likely relevant during early lipid-inflammatory and microcalcific phases, but may also contribute to progression in selected patients with established disease.

This distinction has practical implications for study design. A trial that lowers Lp(a) particle concentration should reduce the particle and its OxPL burden, but the most predictive biomarker may not be Lp(a) mass alone. Future studies should consider molar Lp(a), OxPL-apoB, ATX-Lp(a), apo(a) isoform size, LDL-C corrected for Lp(a)-cholesterol, apoB, inflammatory markers and valve-imaging endpoints. Figure 1 summarizes the proposed Lp(a)-OxPL-ATX pathway in CAVS.

An additional analytical challenge is that Lp(a) mass and particle number are not interchangeable. Because apo(a) isoform size varies, the same mass concentration can represent different particle numbers. For this reason, molar units (nmol/L) are preferred when available, and fixed conversion between mg/dl and nmol/L is discouraged (9, 10). This issue is not trivial in CAVS research because misclassification can dilute associations, obscure thresholds and complicate comparisons across cohorts.

## 5 Genetic and epidemiological evidence

The strongest argument for Lp(a) as a causal factor in CAVS comes from genetics. In a landmark genome-wide association study, variation at the LPA locus was associated with aortic valve calcification and incident aortic stenosis (16). The association was clinically plausible, biologically coherent and subsequently supported by population studies in which elevated Lp(a) and LPA genotypes predicted incident aortic stenosis (17, 18). Broader genome-wide association studies have identified additional aortic stenosis loci, highlighting the polygenic nature of the disease but preserving the central relevance of lipid biology (19, 53).

Mendelian randomization provides a useful framework for distinguishing association from causation. Because Lp(a) concentrations are largely determined at conception, genetic instruments that raise Lp(a) mimic lifelong exposure and are less susceptible to confounding or reverse causation than conventional observational analyses. In this context, the association between genetically predicted Lp(a) and CAVS is especially persuasive (16–19). Similarly, genetic evidence implicating LDL-C and apoB-containing particles suggests that cumulative lipid exposure matters for valve disease, even if conventional LDL-C lowering late in the disease course has not proven effective (44–49).

Epidemiological studies show that higher Lp(a) concentrations are associated with aortic valve calcification, clinically recognized aortic stenosis and aortic valve replacement (17, 28–30, 60). The Copenhagen General Population Study has contributed substantially to the evidence connecting Lp(a) with both valve calcification and overt valve disease (17, 28). Studies in multiethnic cohorts show that Lp(a) associations with valve calcification may be relevant across ancestries, although absolute Lp(a) distributions and risk calibration differ by ancestry (29, 30). The available evidence is summarized by domain in Table 1.

One clinically important issue is whether Lp(a) is more strongly linked to disease initiation than progression. Some studies suggest that Lp(a) is robustly associated with prevalent or incident aortic valve calcification but less consistently associated with calcium-score progression once calcification is present (26, 27). This distinction may reflect biology: Lp(a)-OxPL pathways may be particularly important during early inflammatory and microcalcific phases, whereas advanced stenosis may be driven increasingly by baseline calcium burden, valve anatomy, renal function, fibrosis and mechanical stress. However, haemodynamic progression data are heterogeneous and should not be oversimplified.

## 6 Lp(a), oxidized phospholipids and valvular calcification activity

The Lp(a)-OxPL axis provides a mechanistic bridge between inherited lipid risk and active valve calcification. Other lipid indices, including the ApoB/ApoA-I ratio, have also been associated with faster haemodynamic progression (59). In patients with aortic stenosis, Lp(a) and OxPL have been associated with faster haemodynamic progression, increased valve calcification activity and tissue evidence of osteogenic transformation (21, 22). Capoulade and colleagues (22) showed that OxPL-apoB and Lp(a) were associated with faster progression of peak aortic jet velocity and higher risk of aortic valve replacement or death in mild-to-moderate aortic stenosis. Zheng and colleagues (21) extended these findings by integrating clinical, imaging and tissue data, showing that Lp(a) and OxPL were related to valvular calcification activity and biological pathways promoting osteogenic activity.

Imaging has been critical because echocardiography captures haemodynamic consequences, whereas computed tomography (CT) and positron emission tomography (PET) provide insight into calcific burden and calcification activity. CT aortic valve calcium scoring quantifies established mineral burden and improves assessment of disease severity in selected patients, especially when echocardiographic parameters are discordant (7, 8, 43, 54, 55). 18F-sodium fluoride PET can identify active microcalcification before macroscopic calcification is fully apparent, potentially allowing earlier detection of biologically active disease (23, 56).

Not all studies show the same relationship between Lp(a) and progression. Kaiser and colleagues reported that Lp(a) was associated with the onset of aortic valve calcification but not clearly with calcium-score progression once calcification had developed (26), whereas other cohorts found associations with valve calcification activity, CT calcium progression or haemodynamic progression (21, 22, 57). These differences may reflect distinct endpoints, disease stages, follow-up duration, Lp(a) assays, thresholds, baseline calcium burden and adjustment strategies. Calcium-score progression and haemodynamic progression are related but not identical processes; each may be influenced differently by Lp(a), established mineral burden and valve mechanics.

Other lipid mediators may modify the Lp(a)-CAVS relationship. Oxylipins, including prostaglandins and other eicosanoid-derived mediators, are bioactive products of polyunsaturated fatty-acid oxidation that can regulate inflammation, platelet activation, vascular tone and resolution biology. Recent human valve-tissue work identified multiple oxylipins elevated in severe aortic valve stenosis, and experimental data suggest that pro-resolving lipid mediators may influence valvular inflammation (67, 68). These findings do not diminish the role of Lp(a), but they indicate that OxPL biology sits within a broader network of lipid mediators that may help determine whether valve injury resolves, progresses to fibrosis, or advances to mineralization.

## 7 Does Lp(a) accelerate haemodynamic progression?

Whether Lp(a) accelerates haemodynamic progression of established CAVS is clinically important because it determines whether Lp(a)-lowering therapy should be tested for slowing stenosis, not merely preventing disease. Individual cohort studies have produced mixed results, partly due to differences in Lp(a) assays, thresholds, follow-up duration, disease stage, imaging modality, baseline calcium burden and statistical adjustment.

A 2024 systematic review and meta-analysis synthesized five longitudinal studies including patients with CAVS and found that those in the highest Lp(a) category had faster progression of peak aortic jet velocity and mean transvalvular gradient than those in the lowest category (57). A 2025 analysis of patients with CAVS in the UK Biobank further suggested that Lp(a) levels of 125 nmol/L or higher were associated with higher risk of future aortic valve replacement and composite valvular or cardiovascular outcomes (20). Together, these data strengthen the rationale for valve-specific Lp(a) trials.

However, the evidence remains imperfect. Many studies are relatively small, use different Lp(a) units, lack standardized apo(a) isoform assessment and include heterogeneous disease stages. Some cohorts examine CT calcium progression, whereas others examine haemodynamic progression. Calcium burden is a powerful determinant of future progression; therefore, failure to account for baseline calcium may exaggerate or obscure Lp(a) associations. Conversely, adjustment for baseline calcium may understate the importance of Lp(a) if Lp(a) helped create the baseline calcium burden in the first place.

The apparent discrepancy between strong associations with disease onset and more variable associations with progression should be interpreted as a research opportunity rather than a contradiction. Lp(a)-mediated risk may operate across phases but with different dominant mechanisms. During early disease, Lp(a) may promote lipid deposition, OxPL-driven inflammation and microcalcification. During later disease, it may continue to contribute but compete with established calcium burden, valve geometry, inflammation, fibrosis, renal-mineral biology and haemodynamic stress as determinants of progression.

## 8 Clinical measurement of Lp(a) in patients with aortic stenosis

**Figure 2:**
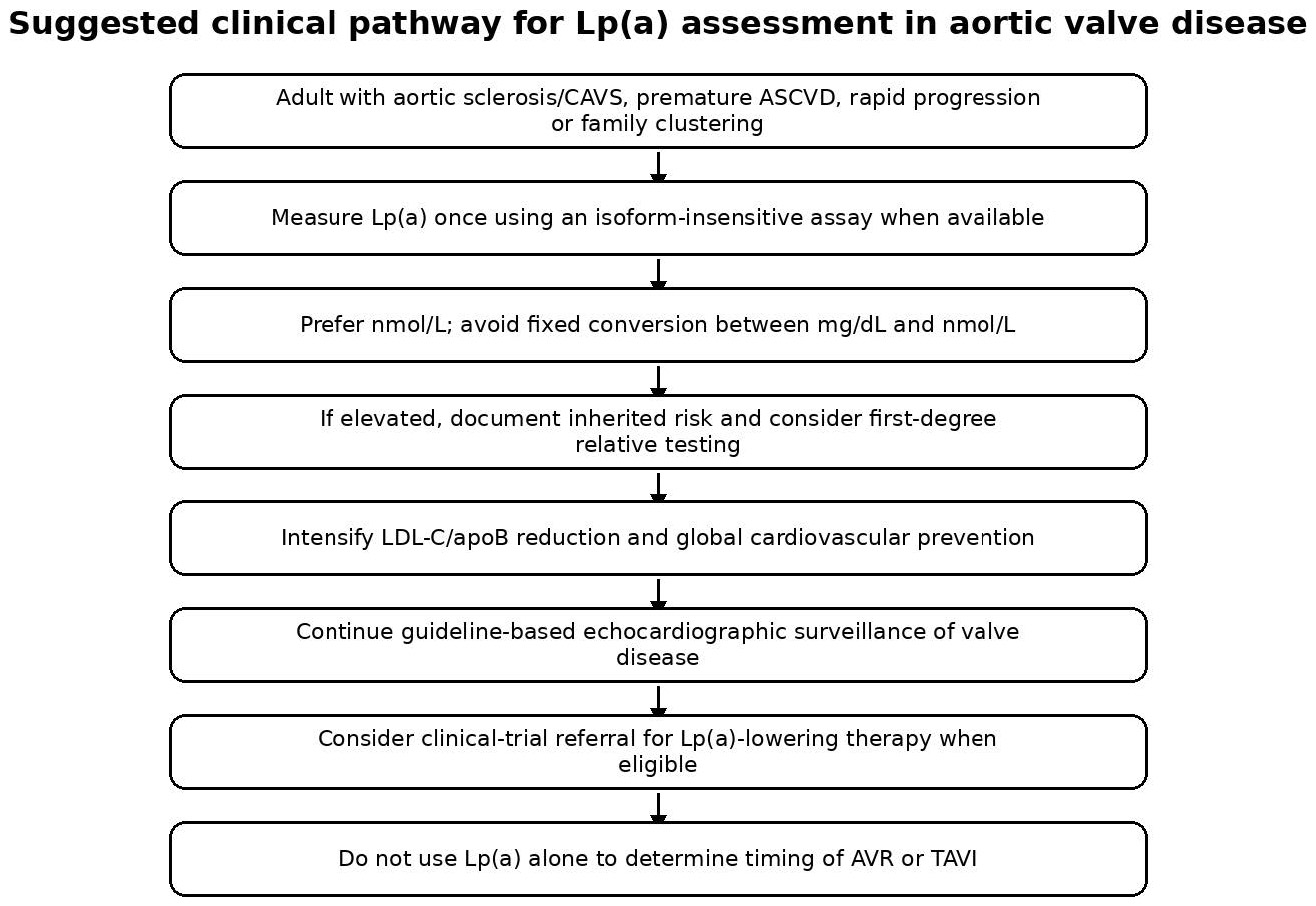
Suggested clinical pathway for Lp(a) assessment in patients with aortic valve disease. Lp(a) measurement is clinically useful today for inherited risk detection and cardiovascular prevention. Its role in selecting patients for valve-specific pharmacological therapy remains investigational.

Contemporary lipid societies increasingly support measuring Lp(a) at least once in adulthood, particularly because Lp(a) is genetically determined, relatively stable and not captured by the standard lipid panel (9–13). From a valvular perspective, measurement is especially reasonable in patients with premature CAVS, severe aortic stenosis without conventional risk factors, family history of aortic stenosis or premature ASCVD, concomitant premature coronary, cerebrovascular or peripheral artery disease, and unexpectedly rapid valve progression. Figure 2 proposes a pragmatic clinical pathway.

Several practical issues must be emphasized. First, Lp(a) may be reported in mg/dl or nmol/L, but conversion using a fixed factor is discouraged because apo(a) isoform size alters particle mass (9, 10). Second, thresholds are not identical across guidelines, but values around 125 nmol/L or 50 mg/dl are commonly considered risk-enhancing, whereas very high concentrations identify individuals with substantially elevated lifetime risk (9, 10, 13). Third, Lp(a) should not be interpreted in isolation. Age, sex, ancestry, LDL-C, apoB, coronary calcium, family history, kidney disease, inflammatory status, valve anatomy and baseline valve calcium all shape absolute risk.

In routine practice, Lp(a) measurement currently has three main consequences. It improves risk communication, supports cascade testing in relatives and justifies aggressive management of modifiable cardiovascular risk factors, especially LDL-C and apoB. In patients with CAVS, it may also encourage careful longitudinal follow-up and referral to centers participating in Lp(a)-lowering trials when appropriate. It should not replace standard valve assessment. Decisions about aortic valve replacement or transcatheter aortic valve implantation must continue to depend on symptoms, echocardiographic severity, ventricular function, haemodynamic response and heart-team evaluation (7, 8).

## 9 Assay standardization, thresholds and the Lp(a)-CAVS phenotype

Implementing Lp(a) measurement in CAVS is deceptively simple. The test is ordered like a lipid measurement, but interpretation requires attention to assay design, reporting units and clinical context. The most important analytical principle is that Lp(a) is a heterogeneous particle. The apo(a) component varies widely in kringle IV type 2 repeat number, and mass-based assays can be influenced by apo(a) isoform size. Modern assays should therefore be isoform-insensitive where possible, and results reported in nmol/L are generally preferred because they better approximate particle concentration (9, 10).

Thresholds also require nuance. For ASCVD, many guidelines and consensus statements treat approximately 50 mg/dl or 125 nmol/L as a risk-enhancing range, with progressively higher lifetime risk at higher levels (9–13). Whether the same threshold should be used for CAVS is not proven. Genetic studies support a likely causal relationship, but the dose-response curve for valve initiation, progression and valve replacement may not be identical to that for myocardial infarction. The 125 nmol/L threshold is clinically practical and increasingly used in contemporary studies, including valvular outcomes research (20), but it should be considered a working threshold rather than a final biological boundary.

The concept of an Lp(a)-CAVS phenotype may be useful but should be presented cautiously. Such patients may present with premature aortic stenosis, family clustering of valve disease, coexisting premature ASCVD, high Lp(a) concentrations, elevated OxPL-apoB or ATX-Lp(a) where available, CT-detected valve calcium and active microcalcification on 18F-sodium fluoride PET (16–23, 61–65). This phenotype could enrich future clinical trials and improve biological interpretation, but it is not yet a validated diagnostic category and should not be used alone to make intervention decisions.

Clinicians should also avoid overinterpreting a normal LDL-C level as evidence against lipid-mediated valve biology. Lp(a)-associated cholesterol may be included within measured LDL-C depending on the assay, and standard lipid panels do not identify high Lp(a) particle burden. ApoB can be helpful for quantifying total atherogenic particle exposure, but apoB does not distinguish LDL particles from Lp(a) particles. For research studies, the most informative lipid phenotype will likely include LDL-C corrected for Lp(a)-cholesterol when possible, apoB, Lp(a) in nmol/L, and inflammatory, OxPL or ATX biomarkers where available (14, 15, 21, 52, 65).

## 10 Why conventional lipid and mineral therapies failed in established disease

Large randomized trials of statin-based lipid lowering did not slow progression of established calcific aortic stenosis (44–46). These trials are sometimes interpreted as evidence against lipid causality in CAVS, but that conclusion is too broad. Several alternative explanations are plausible. First, trial participants generally had established stenosis, often with substantial calcification at baseline. By that stage, the disease may be driven by self-propagating calcific and mechanical pathways less responsive to lipid lowering. Second, statins reduce LDL-C but do not meaningfully reduce Lp(a) and may modestly increase it in some settings. Third, CAVS may require intervention earlier in the disease continuum, before macroscopic calcification dominates. Fourth, LDL-C lowering remains important for ASCVD outcomes in patients with CAVS even if it does not slow valve obstruction.

Other pharmacological strategies targeting mineral metabolism have also failed to demonstrate clear benefit in established disease. In a randomized trial, denosumab or alendronic acid did not slow aortic stenosis progression (58). Together, these results suggest that treating advanced calcific valve disease pharmacologically is difficult. The opportunity for Lp(a)-lowering therapy may therefore lie in prevention, early calcific disease or selected patients with elevated Lp(a) and demonstrably active valve calcification.

A useful analogy is coronary atherosclerosis, in which the benefit of lipid lowering depends on cumulative exposure and time. Waiting until severe valvular obstruction has developed may be analogous to trying to prevent myocardial infarction only after extensive plaque burden and repeated events. In CAVS, the therapeutic trial may need to begin at aortic sclerosis, early CT-detected valve calcification or mild-to-moderate stenosis in patients with high Lp(a), rather than at severe disease. Nevertheless, this remains a hypothesis requiring prospective testing.

## 11 Emerging Lp(a)-lowering therapies

Until recently, clinicians had no potent, specific and scalable therapy for Lp(a). Niacin lowers Lp(a) modestly but did not improve cardiovascular outcomes when added to statin-based therapy in large trials and has tolerability concerns (69, 70). PCSK9 inhibitors reduce Lp(a) by approximately 20%−30% in many patients and reduce ASCVD events, but they were developed primarily for LDL-C lowering and have not been tested specifically for CAVS prevention or progression (71). Lipoprotein apheresis can reduce Lp(a) acutely and may be considered in selected patients with very high Lp(a) and progressive cardiovascular disease despite optimal risk-factor management, but it is resource-intensive and impractical as a broad CAVS prevention strategy (72). Table 2 summarizes therapeutic strategies relevant to CAVS.

**Table 2:** Lp(a)-lowering and related therapeutic strategies: implications for CAVS.

Strategy	Mechanism	Approximate Lp(a) effect	Evidence status	Relevance to CAVS
Lifestyle intervention	Global cardiovascular prevention	Minimal direct reduction	Recommended for global risk, not Lp(a)-specific	Important for ASCVD risk; unlikely to change Lp(a)-mediated valve biology alone
Statins	LDL-C reduction via HMG-CoA reductase inhibition	Little reduction; may modestly increase Lp(a)	Failed to slow established CAVS in randomized trials (44–46)	Continue when indicated for ASCVD risk; not a valve-specific therapy
Niacin	Broad lipid effects	Modest reduction	No incremental outcome benefit and tolerability concerns (69, 70)	Not recommended as valve-specific Lp(a) therapy
PCSK9 inhibitors	LDL receptor pathway; LDL-C reduction	Moderate Lp(a) lowering	ASCVD outcome benefit; no dedicated CAVS trial (71)	May reduce global risk in high-risk patients; valve benefit unproven
Lipoprotein apheresis	Extracorporeal removal of apoB lipoproteins	Large acute reduction with rebound	Selected patients with very high Lp(a) and progressive ASCVD (72)	Impractical as broad CAVS prevention
Pelacarsen	Antisense inhibition of apo(a) synthesis	Large dose-dependent reduction	Phase 2 published; outcome trials ongoing (32, 33)	Potential candidate for valve prevention/progression trials
Olpasiran	siRNA inhibition of apo(a) synthesis	Very large and sustained reduction	Phase 2 and extension data (34, 35)	Strong biological candidate; valvular endpoints needed
Zerlasiran	siRNA inhibition of apo(a) synthesis	Very large reduction with intermittent dosing	Phase 1 and phase 2 published (36–38)	Durable lowering could suit long CAVS natural history
Lepodisiran	Extended-duration siRNA targeting apo(a)	Very large and prolonged reduction	Phase 1 and phase 2 published (39, 40)	Infrequent dosing may be attractive for prevention designs
Muvalaplin	Oral inhibitor of Lp(a) assembly	Moderate-to-large reduction	Phase 1 and phase 2 published (41, 42)	Oral therapy could be scalable if efficacy and safety are confirmed

The therapeutic landscape has changed with nucleic-acid and assembly-targeted therapies. Pelacarsen, an antisense oligonucleotide targeting hepatic apo(a) production, produced substantial Lp(a) reductions in patients with cardiovascular disease (32, 33). Olpasiran, an siRNA agent, lowered Lp(a) dramatically in phase 2 studies, with evidence of sustained off-treatment reduction (34, 35). Zerlasiran and lepodisiran, also siRNA-based therapies, have demonstrated large and durable reductions in Lp(a), including extended-duration effects with infrequent dosing (36–40). Muvalaplin, an oral small-molecule inhibitor of Lp(a) assembly, provides a distinct approach by blocking the apo(a)-apoB interaction and has shown meaningful Lp(a) reductions in early trials (41, 42).

These agents create a biologically plausible path toward testing whether Lp(a) lowering can prevent or slow CAVS. However, three cautions are essential. First, reduction in Lp(a) is not equivalent to proven reduction in clinical events or valve progression. Second, valvular trials may require different endpoints than ASCVD trials, including incident CT valve calcification, 18F-sodium fluoride PET activity, peak aortic jet velocity, mean gradient, valve area, time to severe stenosis and need for valve replacement. Third, the optimal population remains uncertain: individuals with high Lp(a) but no valve disease, patients with early aortic sclerosis, patients with mild-to-moderate CAVS, or patients with established stenosis and rapid progression.

## 12 Designing a valve-specific Lp(a)-lowering trial

A decisive test of the Lp(a)-CAVS hypothesis will require trial designs that respect the natural history of valve disease. The first design question is population selection. Enrolling patients with severe, heavily calcified stenosis may be attractive because event rates are high, but biology suggests that the disease may be relatively autonomous at that stage. Enrolling patients with no valve disease but high Lp(a) may be biologically appealing, but required follow-up could be prohibitively long. A practical compromise may be to study patients with high Lp(a), aortic sclerosis or mild-to-moderate CAVS, and evidence of active calcification or elevated baseline valve calcium. Such enrichment could increase event rates while preserving a modifiable disease window.

The second question is endpoint hierarchy. Hard endpoints such as aortic valve replacement, cardiac death or progression to severe stenosis are clinically meaningful but require large samples and long follow-up. Imaging endpoints can provide earlier signals. CT aortic valve calcium scoring is reproducible and closely related to disease burden, while 18F-sodium fluoride PET identifies active microcalcification and may respond earlier to biological intervention (23, 43, 54–56). Echocardiographic endpoints such as annualized peak velocity, mean gradient and valve area remain clinically familiar and directly linked to patient management, but they may be noisier and influenced by flow state.

The third question is whether Lp(a) lowering should be tested as monotherapy or as part of intensive apoB reduction. Genetic data suggest that Lp(a) and LDL/apoB pathways both matter for CAVS (47–49). From a public health perspective, patients with high Lp(a) should not wait for a valve-specific indication before receiving evidence-based ASCVD prevention. Trial protocols should therefore define background LDL-C and apoB targets clearly. This may reduce confounding and help determine whether residual valve progression is specifically attributable to Lp(a)-mediated pathways.

Finally, trial interpretation should incorporate biomarkers that distinguish mechanistic pathways. If Lp(a) lowering reduces OxPL-apoB, ATX-Lp(a), PET microcalcification and later haemodynamic progression, the mechanistic inference would be stronger than if only Lp(a) concentration changes. Conversely, persistent progression despite deep Lp(a) lowering would not necessarily refute Lp(a) causality for disease initiation; it could indicate that the intervention was too late, that non-Lp(a) pathways dominate advanced disease, or that additional anti-inflammatory, anti-fibrotic or anti-calcific strategies are needed.

## 13 Proposed clinical approach and research agenda

A pragmatic approach is to integrate Lp(a) into CAVS care without overpromising therapeutic implications. In adults with aortic sclerosis or CAVS, Lp(a) measurement can identify inherited risk, inform family screening and motivate aggressive management of modifiable risk factors. In patients with elevated Lp(a), clinicians should optimize LDL-C, apoB, blood pressure, diabetes control, smoking cessation and overall cardiovascular prevention. This is particularly important because patients with CAVS frequently have concomitant atherosclerotic disease and may die from ASCVD events as well as from valve disease.

For the valve itself, management should continue to follow established echocardiographic surveillance intervals and guideline criteria for intervention (7, 8). Elevated Lp(a) alone should not trigger valve replacement, nor should it replace standard assessment of symptoms, left ventricular function, valve hemodynamics and anatomic suitability for surgical or transcatheter treatment. However, in a patient with high Lp(a), early valve calcification and evidence of rapid progression, closer follow-up may be reasonable, especially if other risk factors are present.

A patient-facing implementation strategy is also needed. Elevated Lp(a) is usually inherited, so the result often has implications beyond the individual patient. Explaining that Lp(a) is not primarily a lifestyle marker can reduce stigma, while emphasizing that modifiable risk factors still matter. First-degree relatives may benefit from testing, particularly when there is premature ASCVD, unexplained aortic stenosis or very high Lp(a) in the proband. In clinical records, Lp(a) should be documented as a stable lifelong risk factor rather than repeatedly measured without a clear reason. Repeat testing may be useful when a different assay is used, when results are discordant with the clinical phenotype, or when a patient starts an investigational or approved Lp(a)-lowering therapy.

Several questions should guide the next phase of research. First, what is the most clinically useful Lp(a) threshold for CAVS risk? The threshold for ASCVD may not be identical to the threshold for valve disease, and risk may depend on apo(a) isoform, OxPL content, ancestry and baseline valve calcium. Second, when should therapy begin? If Lp(a) is most important during initiation, trials in established moderate-to-severe stenosis may underestimate benefit. Third, what is the best endpoint? Valve replacement is clinically meaningful but may require large, long studies. Imaging endpoints could provide earlier biological signals but require validation as surrogates. Fourth, how should Lp(a)-lowering therapy be combined with intensive LDL-C or apoB lowering? Given genetic evidence implicating apoB-containing particles broadly, combination prevention may be more effective than targeting Lp(a) alone.

Equity considerations also matter. Lp(a) distributions vary by ancestry, and some populations with high Lp(a) have historically been underrepresented in lipid and valve studies. Future CAVS trials should ensure adequate representation across sex, ancestry, socioeconomic status and valve phenotype, including bicuspid and tricuspid aortic valves. They should also address practical implementation: assay standardization, reporting units, family testing, patient education and cost-effective selection of candidates for expensive therapies.

## 14 Conclusions

Lp(a) is one of the most compelling lipid-related factors in calcific aortic stenosis. It integrates inherited risk, apoB biology, oxidized phospholipid transport, inflammation, autotaxin-lysophosphatidic acid signaling and osteogenic valve calcification. Evidence from genetics, population cohorts, valve imaging, tissue studies and longitudinal progression analyses supports a biologically coherent and clinically relevant relationship. However, the field remains at an inflection point: Lp(a) is measurable today, but specific Lp(a)-lowering therapies are still awaiting definitive valvular outcome evidence.

For clinicians, the immediate message is practical and cautious. Lp(a) should be measured at least once in adulthood according to contemporary lipid guidance and should be considered in patients with premature or unexplained CAVS, rapid valve progression, family history of aortic stenosis or coexisting premature ASCVD. Elevated Lp(a) should prompt comprehensive cardiovascular risk reduction and family awareness. It should not be used alone to determine timing of AVR or TAVI.

For researchers, the central question is whether lowering Lp(a) can change the natural history of valve disease. If effective Lp(a)-lowering therapy prevents CAVS or delays progression in selected patients, CAVS could become one of the first common valvular diseases in which inherited lipid risk is translated into targeted medical prevention. Until that evidence is available, the most defensible position is to recognize Lp(a) as a plausible causal contributor and risk marker while maintaining clinical restraint.
